# Recurrent TFE3‐Rearranged Renal Cell Carcinoma Responding to Pembrolizumab Plus Lenvatinib After Postoperative Pembrolizumab: A Case Report

**DOI:** 10.1002/iju5.70280

**Published:** 2026-10-19

**Authors:** Masashi Fujimoto, Tomoyasu Sano, Yuta Sano, Satoshi Inoue, Kazuna Matsuo, Tomokazu Kimura, Shohei Ishida, Kenji Zennami, Yoshihisa Matsukawa, Shusuke Akamatsu

**Affiliations:** ^1^ Department of Urology Nagoya University Graduate School of Medicine Nagoya Aichi Japan

**Keywords:** Lenvatinib, pembrolizumab, renal cell carcinoma, TFE3, translocation

## Abstract

**Introduction:**

TFE3‐rearranged renal cell carcinoma (RCC) is rare, and evidence guiding systemic therapy is limited.

**Case Presentation:**

A 25‐year‐old man with gross hematuria had a left renal tumor with hilar lymph node metastasis. Robot‐assisted radical nephrectomy and hilar lymph node dissection were performed. Histology, immunohistochemistry, and fluorescence in situ hybridization confirmed TFE3‐rearranged RCC, pT3aN1. Postoperative pembrolizumab was started because of the high risk of recurrence. Eight months after surgery, lung and retroperitoneal metastases developed. Lenvatinib was added to ongoing pembrolizumab. The metastatic lesions decreased within one month, and a partial response according to Response Evaluation Criteria in Solid Tumors has been maintained for ten months.

**Conclusion:**

This case suggests that adding a vascular endothelial growth factor receptor tyrosine kinase inhibitor to pembrolizumab may be a therapeutic option for recurrent TFE3‐rearranged RCC, although longer follow‐up and molecular characterization are needed.

## Keynote Message

This case suggests that adding a tyrosine kinase inhibitor to ongoing pembrolizumab may be a promising treatment strategy for selected patients with TFE3‐rearranged renal cell carcinoma. Although this finding is based on a single case, it highlights a potential treatment option for this rare malignancy. Accumulating similar cases will help clarify the optimal treatment sequence and improve patient outcomes.

## 1 Introduction

TFE3‐rearranged renal cell carcinoma (RCC) is an uncommon molecularly defined RCC characterized by gene fusions involving TFE3. It is diagnosed more frequently in children and young adults than in older adults, but adult cases may be underrecognized because the tumor can mimic clear cell RCC or papillary RCC. The disease can behave aggressively, particularly when it presents with nodal or distant metastasis. Because prospective data are scarce, the optimal systemic treatment for metastatic TFE3‐rearranged RCC remains uncertain. Immune checkpoint inhibitor (ICI)‐based combinations have recently shown activity in non‐clear‐cell RCC and in small TFE3‐rearranged RCC cohorts. Here, we report a young man with recurrent TFE3‐rearranged RCC that responded after lenvatinib was added to pembrolizumab.

## 2 Case Presentation

A 25‐year‐old man presented with gross hematuria. Contrast‐enhanced computed tomography (CT) revealed a 62 × 55 mm heterogeneously enhancing tumor in the left kidney with left renal hilar lymphadenopathy, and the clinical stage was cT3aN1M0 (Figure 1a,b). The patient underwent robot‐assisted radical nephrectomy with hilar lymph node dissection.

**FIGURE 1:**
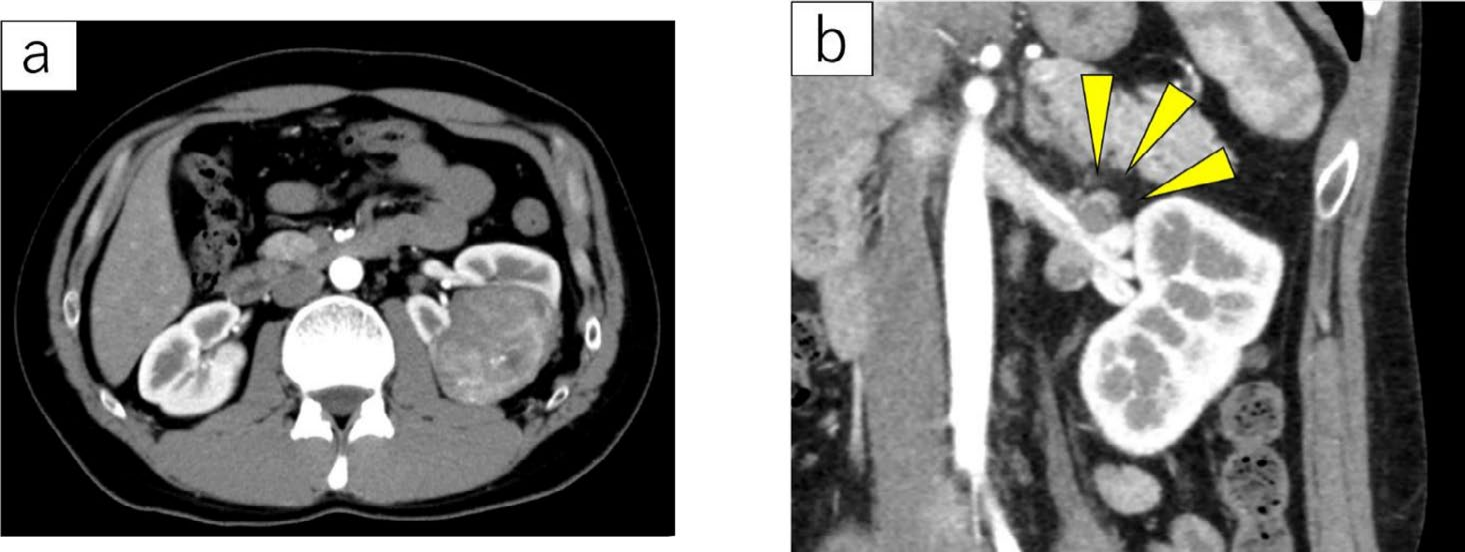
Preoperative contrast‐enhanced computed tomography. (a) A heterogeneously enhancing left renal tumor. (b) Enlarged left renal hilar lymph nodes (arrowheads).

Histopathological examination demonstrated RCC of the left kidney, with a pathological stage of pT3aN1 and WHO/ISUP Grade 2. Hematoxylin and eosin staining showed heterogeneous architectural patterns, including papillary, alveolar, and cystic structures, and tumor cells with clear to pale eosinophilic cytoplasm (Figure 2). Immunohistochemistry showed positivity for TFE3 and alpha‐methylacyl‐CoA racemase, and negativity for c‐kit and cytokeratin 7. Fluorescence in situ hybridization confirmed TFE3 gene rearrangement. Based on these findings, the tumor was diagnosed as TFE3‐rearranged RCC.

**FIGURE 2:**
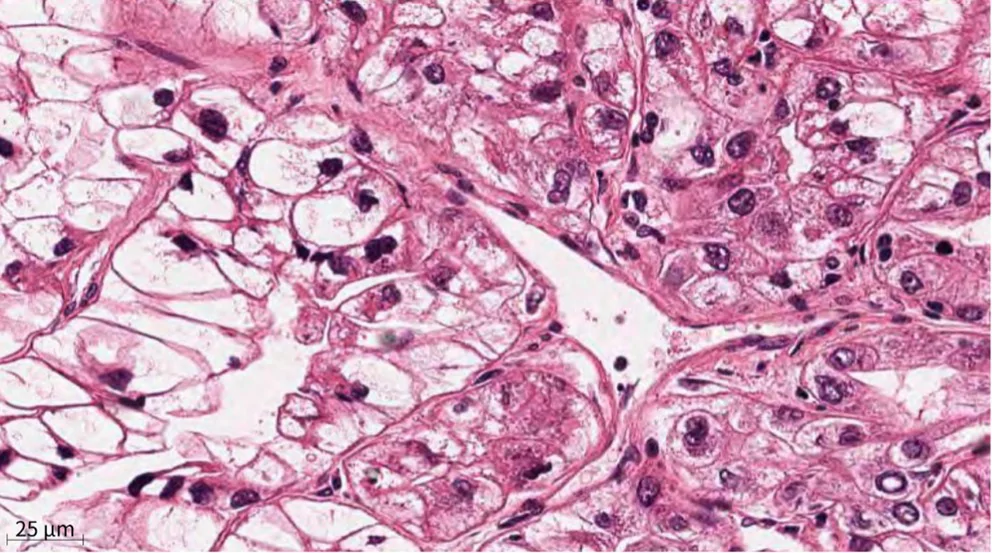
Hematoxylin and eosin staining showing tumor cells with clear to pale eosinophilic cytoplasm in alveolar architecture. Scale bar = 25 μm.

Given the high risk of recurrence, postoperative adjuvant pembrolizumab was initiated. Eight months after surgery, follow‐up CT revealed metastatic lesions in the lung and retroperitoneum (Figure 3a,b). Lenvatinib was therefore added to ongoing pembrolizumab. A reduction in the pulmonary and retroperitoneal lesions was observed one month later (Figure 3c,d). The patient achieved a partial response according to the Response Evaluation Criteria in Solid Tumors, and the response has been maintained for ten months.

**FIGURE 3:**
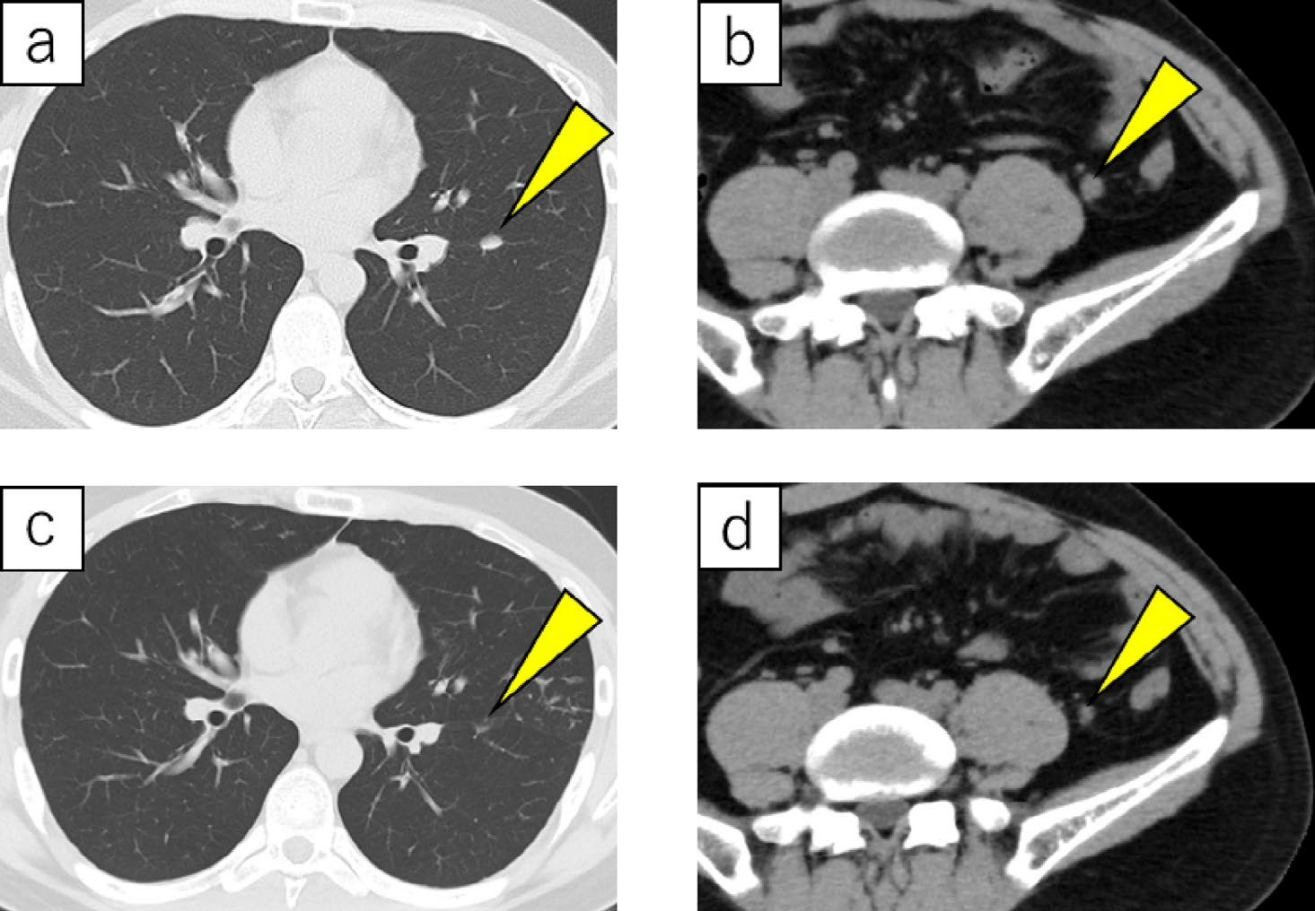
Computed tomography at recurrence and after treatment. (a, b) Lung and retroperitoneal metastatic lesions eight months after surgery. (c, d) Marked reduction of the metastatic lesions one month after lenvatinib was added to pembrolizumab.

## 3 Discussion

TFE3‐rearranged renal cell carcinoma (RCC) is classified as MiT family translocation RCC and is characterized by gene fusions involving TFE3 on Xp11.2 [1, 2]. This tumor accounts for a substantial proportion of pediatric RCC but is uncommon in adults, and its true incidence may be underestimated because morphology often overlaps with clear cell RCC, papillary RCC, or other eosinophilic renal tumors [2, 3, 4]. Adult cases may show aggressive behavior, including lymph node involvement, local invasion, and distant metastasis. Therefore, recognition of this entity is clinically relevant not only for diagnosis but also for treatment selection and follow‐up.

The present case has two important implications. First, it highlights the diagnostic importance of confirming TFE3 rearrangement in young patients or in renal tumors with unusual morphology. The tumor in our case showed heterogeneous architectural patterns and tumor cells with clear to pale eosinophilic cytoplasm. These findings raised the possibility of TFE3‐rearranged RCC, but morphology alone was insufficient for a definitive diagnosis. TFE3 immunohistochemistry and fluorescence in situ hybridization confirmed the diagnosis. Accurate classification is important because TFE3‐rearranged RCC is biologically distinct from conventional clear cell RCC and because evidence for systemic therapy in this rare subtype remains limited.

Adjuvant pembrolizumab was individualized for pT3aN1 disease; KEYNOTE‐564 supports adjuvant use in high‐risk clear‐cell, not TFE3‐rearranged, RCC [5].

Second, this case suggests that an immune checkpoint inhibitor (ICI)‐tyrosine kinase inhibitor (TKI) strategy may have activity even after recurrence during pembrolizumab therapy. Systemic treatment for metastatic TFE3‐rearranged RCC has not been standardized. Retrospective studies have reported variable outcomes with vascular endothelial growth factor receptor TKIs, mammalian target of rapamycin inhibitors, and ICI monotherapy [6, 7, 8]. These inconsistent results probably reflect the rarity of the disease, the small number of treated patients, differences in fusion partners, and the biological heterogeneity of MiT family translocation RCC.

More recently, ICI‐based combination therapy has emerged as a promising strategy for non‐clear‐cell RCC. In KEYNOTE‐B61, pembrolizumab plus lenvatinib demonstrated clinically meaningful activity in advanced non‐clear‐cell RCC, with responses also observed in patients with translocation histology [9, 10]. Although the number of patients with TFE3‐rearranged RCC in such studies is small, these data provide a rationale for combining immune activation with antiangiogenic therapy. Lenvatinib inhibits several kinases, including vascular endothelial growth factor receptors and fibroblast growth factor receptors, and may contribute to tumor control by suppressing angiogenesis and modifying the tumor microenvironment. This mechanism may be particularly relevant in selected TFE3‐rearranged RCCs with angiogenesis‐related molecular features. Lenvatinib may enhance PD‐1 blockade through macrophage, interferon, and CD8+ T‐cell modulation [11, 12].

A notable feature of the present case is that recurrence occurred during postoperative pembrolizumab, whereas tumor reduction was observed after lenvatinib was added. Thus, this case is not simply a first‐line ICI‐TKI response but may represent clinical activity of lenvatinib add‐on therapy after insufficient disease control with pembrolizumab alone. When recurrence develops during postoperative ICI, treatment selection is clinically challenging, particularly in rare non‐clear‐cell histologies for which prospective evidence is scarce. The early radiographic reduction within one month and the ongoing partial response also suggest that clinically meaningful disease control can be achieved in selected patients. The favorable response may have resulted from direct antiangiogenic effects of lenvatinib, enhancement of antitumor immunity through tumor microenvironment modulation, or both. However, this interpretation remains speculative because this is a single case and because the contribution of continuing pembrolizumab cannot be separated from the effect of lenvatinib. KEYNOTE‐146 reported activity after prior ICI [13]; surveillance until recurrence was also reasonable, and sequence superiority is unknown.

Recent molecular analyses suggest that fusion partners and gene‐expression profiles may influence treatment response in TFE3‐rearranged RCC. In particular, ASPSCR1‐TFE3 fusion has been associated with angiogenesis‐, stroma‐, and proliferation‐related signatures and with favorable outcomes after ICI‐based combination therapy in some cohorts [14, 15]. Unfortunately, RNA sequencing could not be performed in the present case because appropriate tumor samples were unavailable, and the fusion partner could not be identified. This is a limitation of our report. Further accumulation of cases with detailed molecular annotation, treatment sequence information, and long‐term follow‐up is needed to determine which patients with TFE3‐rearranged RCC are most likely to benefit from ICI‐TKI therapy.

## 4 Conclusion

We report a case of recurrent TFE3‐rearranged RCC that achieved an ongoing partial response after lenvatinib was added to pembrolizumab. This case supports considering ICI‐TKI combination therapy for metastatic or recurrent TFE3‐rearranged RCC and highlights the need for molecular characterization in this rare subtype.

## Funding

The authors have nothing to report.

## Consent

Written informed consent was obtained from the patient for publication of this case report and accompanying images.

## Conflicts of Interest

The authors declare no conflicts of interest. Dr. Shohei Ishida is an Editorial Board member of International Journal of Urology and a co‐author of this article. To minimize bias, he was excluded from all editorial decision‐making related to the acceptance of this article for publication.
